# A Novel Truncating Mutation in HOMER2 Causes Nonsyndromic Progressive DFNA68 Hearing Loss in a Spanish Family

**DOI:** 10.3390/genes12030411

**Published:** 2021-03-12

**Authors:** María Lachgar, Matías Morín, Manuela Villamar, Ignacio del Castillo, Miguel Ángel Moreno-Pelayo

**Affiliations:** 1 Servicio de Genética, Hospital Universitario Ramón y Cajal, IRYCIS, Carretera de Colmenar km 9.100, 28034 Madrid, Spain; maria.lachgar95@gmail.com (M.L.); matmorinro@yahoo.es (M.M.); taugen.hrc@salud.madrid.org (M.V.); delcastilloi@hotmail.com (I.d.C.); 2 Centro de Investigación Biomédica en Red de Enfermedades Raras (CIBERER), 28034 Madrid, Spain

**Keywords:** hereditary hearing loss, next-generation sequencing, custom panel, HOMER2, CDC42

## Abstract

Nonsyndromic hereditary hearing loss is a common sensory defect in humans that is clinically and genetically highly heterogeneous. So far, 122 genes have been associated with this disorder and 50 of them have been linked to autosomal dominant (DFNA) forms like DFNA68, a rare subtype of hearing impairment caused by disruption of a stereociliary scaffolding protein (HOMER2) that is essential for normal hearing in humans and mice. In this study, we report a novel HOMER2 variant (c.832_836delCCTCA) identified in a Spanish family by using a custom NGS targeted gene panel (OTO-NGS-v2). This frameshift mutation produces a premature stop codon that may lead in the absence of NMD to a shorter variant (p.Pro278Alafs*10) that truncates HOMER2 at the CDC42 binding domain (CBD) of the coiled-coil structure, a region that is essential for protein multimerization and HOMER2-CDC42 interaction. c.832_836delCCTCA mutation is placed close to the previously identified c.840_840dup mutation found in a Chinese family that truncates the protein (p.Met281Hisfs*9) at the CBD. Functional assessment of the Chinese mutant revealed decreased protein stability, reduced ability to multimerize, and altered distribution pattern in transfected cells when compared with wild-type HOMER2. Interestingly, the Spanish and Chinese frameshift mutations might exert a similar effect at the protein level, leading to truncated mutants with the same Ct aberrant protein tail, thus suggesting that they can share a common mechanism of pathogenesis. Indeed, age-matched patients in both families display quite similar hearing loss phenotypes consisting of early-onset, moderate-to-profound progressive hearing loss. In summary, we have identified the third variant in HOMER2, which is the first one identified in the Spanish population, thus contributing to expanding the mutational spectrum of this gene in other populations, and also to clarifying the genotype–phenotype correlations of DFNA68 hearing loss.

## 1. Introduction

Hearing loss is the most common sensory deficit in humans, affecting around 1 in 1000 newborns. Its prevalence increases with the age up to 6–8% in the adult population, having a strong impact on the individual’s social isolation [1]. Genetic causes account for 50–60% of newborn hearing loss, 30% of which are nonsyndromic forms of deafness [2]. The genetic etiology of hearing impairment is highly heterogeneous. Up to date, 160 nonsyndromic sensorineural hearing loss (NSSNHL) *loci* have been mapped, of which 122 genes have been identified [3]. Sixty-seven of these *loci* and 50 of these genes are associated with autosomal dominant NSSNHL, being, in most cases, rare forms of post-lingual and progressive hereditary hearing impairment.

Autosomal dominant NSSNHL-linked genes encode proteins with a wide variety of functions [4], such as cytoskeleton proteins (*ACTG1*, *DIAPH1*, *PLS1*), adhesion proteins (*GJB2*, *GJB3*, *GJB6*, *TJP2*), motor proteins (myosins), scaffolding proteins (*HOMER2*), extracellular matrix proteins (*TECTA*, *COL11A2*), proteins involved in ion homeostasis, such as ionic channels (*KCNQ4*), transcription factors (*EYA4*, *POU4F3*), and even a microRNA (*MIR96*) [5].

The *HOMER2* gene maps to chromosome 15q24.3 [6] within the DFNA68 critical interval and consists of nine exons. Two human transcript variants have been described (NM_004839.4 and NM_199330.3) as encoding the short isoform 1 (NP_004830.2, 343aa) and the long isoform 2 (NP_955362.1, 354aa) of HOMER2, respectively. HOMER2 belongs to a protein family encompassing three members: HOMER1 (MIM604798), HOMER2/CUPIDIN (MIM604799), and HOMER3 (MIM604800). Like HOMER2, members 1 and 3 of the family have long and short isoforms due to alternative splicing [7]. HOMER family members are scaffolding proteins that play a key role in Ca^2+^ signaling [8,9,10], mostly at the Post-Synaptic-Densities (PSD) [11], where they interact with G-protein coupled metabotropic glutamate receptors (mGluRs) [12] and regulate excitatory signal transduction and receptor plasticity [7]. In their structure, HOMER proteins present a conserved N-terminal domain known as Enabled/vasodilator-stimulated phosphoprotein (Ena/VASP) homology 1 (EVH1) domain [7,13] that binds to proline-rich sequences (i.e. Pro-Pro-x-x-Phe, Pro-x-x-Phe or Leu-Pro-Ser-Ser-Pro, where x represents any amino acid) and a C-terminal domain that consists of a coiled-coil (CC) structure that includes a CDC42 binding domain (CBD) and two Leucine Zipper (LZA and LZB) motifs [7,14]. This C-terminal fragment mediates self-association with other Homer family members [7] and the interaction with the small GTPase CDC42 [15] through its CBD.

In mice, *Homer2* is widespread in the developing and maturing brain [16]. Recently, a study has shown that this gene is also expressed in a wide variety of developing tissues, including tooth, eye, cochlea, salivary glands, olfactory and respiratory mucosae, bone, and taste buds, being highly concentrated at puncta [17]. HOMER2 exhibits overlapping distribution patterns with HOMER1 and HOMER3, although they are distributed at distinct subcellular domains in several cell types [17,18]. Within the inner ear, HOMER2 is expressed in the stria vascularis, the Reissner’s membrane, and the inner and outer hair cells of the organ of Corti, especially in the stereocilia but also in perinuclear puncta and the cytoplasm [17,18]. Mild expression is observed in the vascular endothelium of the cochlea and the spiral ganglion [12]. Mice homozygous for the targeted deletion of *Homer2* display early-onset rapidly progressive hearing loss [18].

*HOMER2* was firstly associated with hearing loss by Azaiez et al. [18] who identified a heterozygous missense variant (p.Arg196Pro) in a European ascent family. A second mutation in *HOMER2* (c.840_840dup; p.Met281Hisfs*9) was identified by Lu et al. [19] by Whole Exome Sequencing (WES) in a Chinese family segregating with hearing loss. Here, we present a third variant in *HOMER2* (c.832_836delCCTCA, p.Pro278Alafs*10, NM_199330.3). This variant represents the second truncating mutation that affects the CBD and lead, as in the previous two families, to post-lingual and progressive hearing loss. This mutation is the first one identified in the Spanish population, thus increasing the mutational spectrum of this gene associated with DFNA68, a rare form of autosomal dominant nonsyndromic progressive hearing loss.

## 2. Patients and Methods

### 2.1. Patients Selection

**Figure 1:**
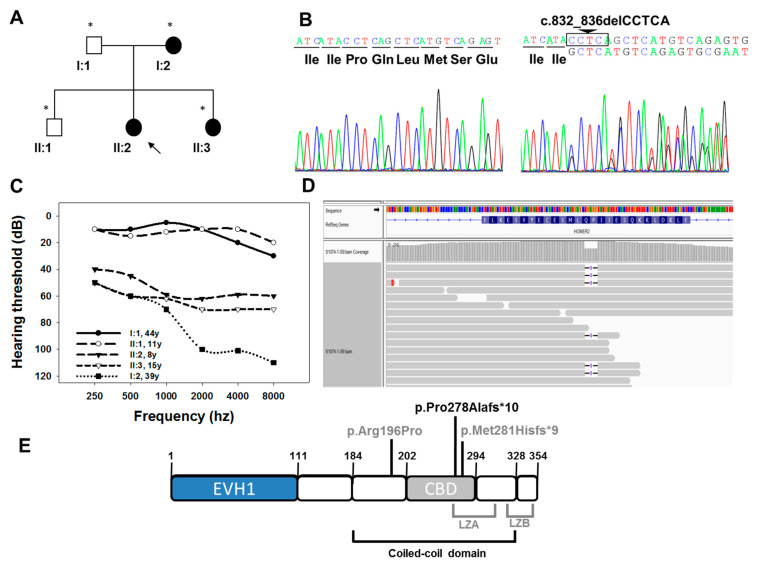
(**A**) Pedigree of the S1074 family indicating the segregation of c.832_836delCCTCA mutation in *HOMER2*. Black symbols indicate affected patients (carrying the mutation in heterozygosis), and white symbols correspond to normal hearing individuals (wild-type for the mutation studied). The subject pointed with an arrow is the index case (studied by OTO-NGS-v2 panel), and the ones marked with an asterisk were analysed by Sanger sequencing for segregation analysis. (**B**) Electropherograms corresponding to the wild-type (left) and mutant (right) sequences of a normal hearing and an affected individual, respectively. (**C**) Audiograms of the S1074 family. The data represented correspond to the average of the audiometric thresholds in both ears. (**D**) Integrative Genomics Viewer (IGV) (Broad Institute) screenshot showing the genomic region corresponding to the c.832_836delCCTCA mutation of *HOMER2* and the translated protein stretch corresponding to exon 8 in the reverse orientation (NP_955362.1, HOMER2 long isoform, amino acid range 292–266), as *HOMER2* is transcribed by using the DNA negative strand. (**E**) Schematic representation of the structure of HOMER2 long isoform (NP_955362.1). The different domains [7,14,21] and the mutations identified so far associated with hearing loss (in grey) are shown. The mutation identified in this work is shown in black. EVH1 (1–111 aa): Enabled/vasodilator-stimulated phosphoprotein (Ena/VASP) homology 1; Coiled-coil domain (184–328 aa); CBD (202–294 aa): CDC42-binding domain. LZA (249–307 aa): Leucine Zipper-A; LZB (322–350 aa): Leucine Zipper-B.

Patients and healthy relatives of family S1074 (Figure 1A) were recruited from the University Hospital Ramón y Cajal (Madrid-Spain). Clinical history ruled out environmental factors as the cause of the hearing loss in the probands, and physical examination did not reveal any evidence of syndromic features. No other clinically significant manifestations, including balance or visual problems, were reported by any of the affected individuals. The hearing level was evaluated through pure tone audiometry. Air conduction thresholds were determined at frequencies ranging from 250 to 8000 Hz according to standard protocols. This study was designed in compliance with the tenets of the Helsinki Declaration, and patient enrolment was approved by the ethics committee and the human research Institutional Review Boards of Hospital Ramón y Cajal (IRB number: 288-17). All participants of the family approved of the study and signed the Informed Consent.

### 2.2. Sample Collection

A peripheral blood sample from each subject of the family S1074 enrolled in the study was collected by venipuncture in 5 mM EDTA tubes and genomic DNA was extracted using Chemagen MSM I (Magnetic Separation Module I, PerkinElmer, Massachusetts, MA, USA) according to the manufacturer’s instructions. DNA was quantified by the fluorometric method Qubit 3.0 Fluorometer (Thermo Fisher Scientific, Massachusetts, MA, USA).

### 2.3. Targeted Next-Generation Sequencing

The index case of the family (II:2) was subjected for the genetic screening for causative hearing-loss mutations by using a custom gene panel, OTO-NGS-v2, designed in our laboratory [20]. As the causative mutation was identified following this approach, whole exome sequencing (WES) on the individual II:2 was not further performed. OTO-NGS-v2 is based on IDT probes capture system that included 117 genes associated with NSSNHL. Sequencing of captured enriched-libraries was done on the Illumina MiSeq (Illumina, Inc., San Diego, CA, USA). The sequence data were mapped against the human genome sequence (build GRCh37/hg19), and data analysis was performed using the Sophia Genetics’ software that enables the single nucleotide variations (SNVs) and the copy number variation (CNV) analysis of the targeted exonic sequences. Variant prioritization was carried out using a custom filtering strategy [20].

### 2.4. Sanger Sequencing

The c.832_836delCCTCA mutation in exon 8 of *HOMER2* [NM_199330.3, long transcript] was verified by Sanger sequencing (Figure 1B). Briefly, a forward and a reverse oligonucleotide were designed for amplification of exon 8 (F-oligo 5′-CGTGCACACATTGGTGATTT-3′ and R-oligo 5′-AAGCAGGAAAATGAGTACCATGA-3′) followed by Sanger sequencing using the BigDye™ Terminator v3.1 Cycle Sequencing Kit (Applied Biosystems, Foster City, CA, USA) according to manufacturer’s directions in an ABI 3730S sequencer (Perkin Elmer, Waltham, MA, USA). The specificity of the primers designed for the amplification and Sanger sequencing of exon 8 of *HOMER2* was confirmed by BlastN (https://blast.ncbi.nlm.nih.gov/; access date: 9 March 2021). We obtained a unique blast hit for the amplimer at chromosome 15 (NC_000015.10; coordinates 82,851,291 to 82,851,310) within the *HOMER2* genomic region. Segregation analysis was performed by checking the presence of the c.832_836delCCTCA mutation in all the affected and unaffected family members.

## 3. Results

### 3.1. Clinical Description of the Family

The clinical history and audiological assessments of the affected members reported a post-lingual bilateral progressive hearing loss consistent with an autosomal dominant inheritance pattern (Figure 1A). Individuals II:2 (8 years old) and II:3 (15 years old) exhibited moderate hearing loss with greater impact on the high frequencies (downsloping profile). Their mother (patient I:2, 39 years old) showed a more severe phenotype, displaying profound hearing loss at frequencies higher than 2000 Hz. The father (I:1, 44 years old) and his healthy son (II:1, 11 years old) showed normal hearing thresholds (Figure 1C) at the time of the study.

### 3.2. Genetic Study

By using the OTO-NGS-v2 panel and Sophia DMM software 338 heterozygous genetic variants were retained in the index case (II:2) of the family, 10 of which were classified as potentially pathogenic in *HOMER2*, *MYO7A*, *TMPRSS3*, *COL11A2*, *GPSM2*, *BDP1*, *EPS8*, *EPS8L2*, *OSBPL2,* and *PCDH15* genes, respectively (Table 1). During the tertiary analysis, 9 variants were discarded. Two of them showed high population frequencies in the Genome Aggregation Database (GnomAD) and were classified as benign variants in ClinVar (*MYO7A*, *COL11A2*). The other 6 variants were associated with recessive forms of deafness (*TMPRSS3*, *GPSM2*, *BDP1*, *EPS8*, *EPS8L2*, and *PCDH15*). The variant c.747A>G (rs1309059934) in *OSBPL2* (DFNA67) was classified by Sophia DMM as potentially pathogenic. However, it resulted in a synonymous protein change (p.Arg249Arg) that was classified as likely benign (BP4, BP7, and PM2) according to the American College of Medical Genetics and Genomics (ACMG) guidelines [22,23]. Furthermore, we confirmed by Sanger sequencing that this mutation did not segregate with the hearing loss in the family as it was detected in the normal-hearing subject II:I.

Finally, a novel mutation in *HOMER2* gene, c.832_836delCCTCA, was identified in the *propositus* of the S1074 family (patient II:2). This variant was identified at exon 8 of *HOMER2* and is supposed to alter the two transcript variants with known reported expression (NM_004839.4, short transcript, and NM_199330.3, long transcript). The variant leads to a frameshift generating a premature stop codon that in the absence of nonsense-mediated decay (NMD) may produce a truncated HOMER2 protein (p.Pro278Alafs*10). This mutation was classified as pathogenic (PVS1, PM2 and PP3) according to the ACMG guidelines [22,23]. The variant was not present in the GnomAD [24], in the Collaborative Spanish Variant Server-CSVS database [25] nor in the Deafness Variation Database (DVD, http://deafnessvariationdatabase.org/; access date: 9 March 2021;) [26,27]. Additionally, we confirmed by Sanger sequencing (Figure 1B) it segregated with the hearing loss in the family. The novel *HOMER2* variant c.832_836delCCTCA has been deposited in ClinVar (accession# SCV001499845). Comparison of the genetic and clinical data of the S1074 family with previously reported cases is made in Table 2.

## 4. Discussion

Hearing loss caused by *HOMER2* mutations is an extremely rare disorder. In this study, we have used OTO-NGS-v2, a custom targeted NGS panel, for the identification of a novel mutation (c.832_836delCCTCA; p.Pro278Alafs*10) in this gene linked to DFNA68 hearing loss. To date, only two different mutations in *HOMER2* have been documented to cause hearing loss. The first mutation was a missense substitution (c.587G>C; p.Arg196Pro; NM_199330.3) that affects a highly conserved residue in the coiled-coil (CC) structure, a region that is required for homo/hetero-multimerization to form tetrameric hubs (in which the CC domains align in a parallel fashion) and for interaction through the CBD with Rho family GTPase proteins like CDC42, a GTPase that mediates actin-turnover [28] and is responsible for planar polarity establishment in hair cells [29,30]. Functional assessment of the p.Arg196Pro in zebrafish strongly suggests that this mutation exerts its effect through a dominant-negative mechanism on wild-type protein by either inhibiting multimerization or competing for partner proteins [18]. The second mutation, c.840_840dup, p.Met281Hisfs*9 (NM_199330.3) was identified in a Chinese family with symmetric ADSNHL [19]. This frameshift variant at the CDC42-binding domain leads to the generation of a premature stop codon supposed to produce a truncated HOMER2 protein of 288 amino acids, with an aberrant protein tail of 8 amino acids from position 281 onwards.

**Figure 2:**
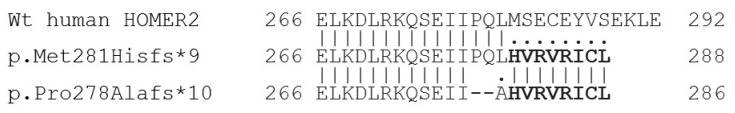
Alignment of the wild-type protein fragment encoded by exon 8 of *HOMER2* long isoform (NP_955362.1) and the truncating mutations in the CDC42-binding domain (CBD) identified in the Chinese (p.Met281Hisfs*9) and Spanish (p.Pro278Alafs*10) families. The amino acid sequence shared between both aberrant protein tails is shown in bold face.

In this work, we have identified a novel frameshift mutation, c.832_836delCCTCA, p.Pro278Alafs*10 in *HOMER2* that represents the second truncating mutation identified in the CDC42-binding domain. The Spanish mutation also creates a premature stop codon that may lead to generate a truncated shorter protein of 286 amino acids with an aberrant tail of 9 amino acids from position 278, thus lacking the canonical C-terminal end. Interestingly, the alignment of both truncated proteins revealed an identical sequence of the last 8 amino acids of the aberrant tails (Figure 2).

Based on the similar effect that the two different frameshift mutations may cause at the protein level, both supposed to truncate the protein at the CDB and resulting in the same aberrant tail, it might be reasonable to suggest that the Chinese and Spanish mutations may share a similar mechanism of pathogenesis. Lu et al. demonstrated that the p.Met281Hisfs*9 mutant protein was less stable than the wild-type protein and it showed an altered subcellular localization in HEK293T and HEI-OC1 cells. Whereas the wild-type proteins were mainly aggregated near the nucleus, the p.Met281Hisfs*9 mutants were more widely distributed throughout the cytoplasm. Furthermore, these authors demonstrated that p.Met281Hisfs*9 showed a decreased ability to oligomerize [19]. It has also been postulated that HOMER2 could play an important role in maintaining stereocilia through its interaction with CDC42 [19], therefore the truncation of HOMER2 in the CBD could eventually prevent its interaction with other HOMER protein family members and with other proteins like CDC42. Indeed, targeted deletion of Cdc42 in murine hair cells causes a progressive hearing loss phenotype that is comparable to the hearing loss phenotype in the Spanish and Chinese families [19]. Another possibility is that the *HOMER2* mutation identified in this study may cause nonsense-mediated decay (NMD), a mechanism that affects the processing of the transcripts at different extent depending on the type of mutation and gene involved as previously reported in other pathologies like Neurofibromatosis I [31]. In this regard, and in contrast to mouse mutants homozygous for the targeted deletion of *Homer2* that display early-onset rapidly progressive hearing loss, mice heterozygous for the targeted deletion of exon 3 in *Homer2* (*Homer^−/+^*) displayed normal hearing levels [18]. It may indicate that a moderate or even low extent NMD might be associated with *HOMER2* frameshift mutations thus suggesting that the pathophysiology of the DFNA68 hearing loss in the Spanish and Chinese families would not be mediated by haploinsufficiency, but by a gain-of-function of the Ct aberrant tail or by a dominant-negative mechanism as it has been postulated for the p.Arg196Pro missense mutation [18]. However, more experiments to detect *HOMER2* mutant transcripts levels by using a gene-editing tool to mimic actual mutation in cell lines or the generation of knock-in murine models are necessary to fully understand the underlying mechanism of pathogenesis linked to DFNA68 frameshift mutations.

Regarding the clinical phenotype, the hearing loss observed in *HOMER2* patients caused by the missense (p.Arg196Pro) and two truncating mutations seems to be quite similar in the three studied families. Affected individuals show progressive hearing loss affecting mainly the high frequencies (downsloping profile) with a typical onset in the first decade of life (7–9 years old), although a more severe phenotype was detected in patients bearing the truncating mutations when age-matched hearing-impaired subjects of the three families were compared. For individuals in a similar age-range, those carrying the missense p.Arg196Pro mutation [18] displayed minor affectation on the entire range of frequencies (i.e., subject IV:2, 8y.o, IV:10, 15y.o, and III:4, 34y.o, European ascent family) than patients carrying the p.Met281Hisfs*9 (subject IV:7, 7y.o; III:10, 34y.o, and III:8, 39y.o in the Chinese family) or the p.Pro278Alafs*10 variant (subject II:2, 8y.o; II:3, 15y.o, and I:2, 39y.o in the Spanish family). The presence of tinnitus or cranial tinnitus, however, has only been displayed by some affected members of the Chinese family and this phenotype was not present in any of the other two families. The identification of other *HOMER2* cases is, therefore, necessary to further establish more accurate genotype–phenotype correlations.

In summary, we have identified a third variant in *HOMER2*; the first one reported in the Spanish population, thus contributing to expanding the mutational spectrum of this gene in other populations. Our study also highlights the importance of using NGS-based diagnostic methods to identify mutations in low-prevalence deafness genes like *HOMER2*, thus helping to improve our knowledge about the pathophysiology of DFNA68 and to define more accurate genotype–phenotype correlations in this disorder.

## Figures and Tables

**Table 1:** Genetic variants with potential pathogenicity identified by SOPHIA DDM software in the index case (II:2) of family S1074.

Gene Transcript	Exon	c.DNA and Protein Alteration	Variant Fraction Coverage (Ref/Alt)	Coding Consequence	Pathogenicity Prediction by SOPHIA DDM	ACMG	ClinVar Rating	GnomAD	*Locus*
*HOMER2* NM_199330	8	c.832_836delCCTCA p.Pro278Alafs*10	34.98% (145/78)	frameshift	Highly Pathogenic	Pathogenic (PVS1, PM2, PP3)	-	0	DFNA68
*MYO7A* NM_001127179	27	c.3515_3536del p.Gly1172*1179del	49.0% (582/306)	No-stop	Highly Pathogenic	Benign (PVS1, BA1, BS2, BP6)	Benign rs111033223	0.391	DFNA11 DFNB2
*TMPRSS3* NM_001256317	8	c.617-3_617-2dupTA p.?	46.73% (421/372)	Splice acceptor	Highly Pathogenic	(PVS1, PP3, BA1, BP6)	Benign rs34966432	0.118	DFNB8 DFNB10
*COL11A2* NM_080679	32	c.2307 + 3G>A p.?	50.43% (402/409)	splice_donor_ +3	Potentially Pathogenic	Benign (BA1, BS2)	Benign rs970901	0.601	DFNA13 DFNB53
*GPSM2* NM_013296	13	c.1572_1574delTTC p.Ser525del	48.55% (278/267)	inframe_3	Potentially Pathogenic	Benign(PP3, BA1, BP3, BP6)	Benign rs35029887	0.291	DFNB82
*BDP1* NM_018429	23	c.5068G>C p.Gly1690Arg	44.54% (335/269)	missense	Potentially Pathogenic	Likely Benign (PM2, BP1, BP4)	- rs193135814	7.48 × 10^−5^	DFNB112
*EPS8* NM_004447	20	c.2230G>A p.Val744Ile	44.34% (359/286)	missense	Potentially Pathogenic	Benign (BS1, BS2, BP1, BP6)	Likely Benign rs77967764	2.06 × 10^−3^	DFNB102
*EPS8L2* NM_022772	9	c.710G>T p.Arg237Leu	50.22% (560/565)	missense	Potentially Pathogenic	VUS (PM2, BP4)	-	7.1 × 10^−6^	DFNB106
*OSBPL2* NM_014835	9	c.747A>G p.Arg249Arg	51.09% (157/164)	synonymous	Potentially Pathogenic	Likely Benign (BP4, BP7, PM2)	- rs1309059934	0	DFNA67
*PCDH15* NM_001142771	21	c.2596G>A p.Val866Met	49.09% (446/430)	missense	Potentially Pathogenic	VUS (PM1, PM2)	Uncertain Significance rs142512524	6.37 × 10^−4^	DFNB23

**Table 2:** Clinical data and classification of the different variants described in *HOMER2* causing hearing loss.

DNA Change	Protein Change	Exon	Origin	Phenotype	Detection Decade	Degree	Audiogram Profile	ACMG Classification	Number of Scores Supporting Pathogenicity Pathogenicity	DVD	CSVS Allele Freq	GnomAD Allele Freq.	References
c.587G>C	p.Arg196Pro	6	European descent	SNHL	1st	Mild-profound	Down-sloping	Uncertain significance (PM2, PP3, PP5, BP1)	20/22	N.A	0	0	Azaiez et al. 2015
c.840_840dup	p.(Met281Hisfs*9)	8	Chinese	SNHL	1st	Moderate-profound	Down-sloping	Pathogenic (PVS1, PM2, PP3, PP5)	N.A	N.A	0	0	Lu et al. 2018
c.832_836delCCTCA	p.(Pro278Alafs*10)	8	Spanish	SNHL	1st	Moderate-profound	Down-sloping	Pathogenic (PVS1, PM2, PP3)	N.A	N.A	0	0	This work

All the variants are named according to NM_199330.3 long transcript and the nomenclature was checked using Mutalyzer 2.0.32. ACMG criteria [22,23]: PVS1 (Pathogenic Very Strong): null variant (nonsense, frameshift, canonical ±1 or 2 splice sites, initiation codon, single or multiexon deletion) in a gene where LOF is a known mechanism of disease. PM2 (Pathogenic Moderate 2): absent from controls (or at extremely low frequency if recessive) in Exome Sequencing Project, 1000 Genomes Project, or Exome Aggregation Consortium. PP3 (Pathogenic Supporting 3): multiple lines of computational evidence support a deleterious effect on the gene or gene product (conservation, evolutionary, splicing impact, etc.). PP5 (Pathogenic Supporting 5): reputable source recently reports variant as pathogenic, but the evidence is not available to the laboratory to perform an independent evaluation. BP1 (Benign Supporting 1): missense variant in a gene for which primarily truncating variants are known to cause disease. The databases GnomAD [24], CSVS [25], and Deafness Variation Database (DVD) [26] were searched on the 20th December 2020. SNHL: sensorineural hearing loss, NA: not available.

## Data Availability

The data presented in this study are openly available in ClinVar (accession# SCV001499845).
